# Metabolomics in Primary Open Angle Glaucoma: A Systematic Review and Meta-Analysis

**DOI:** 10.3389/fnins.2022.835736

**Published:** 2022-05-12

**Authors:** Yizhen Tang, Simran Shah, Kin-Sang Cho, Xinghuai Sun, Dong Feng Chen

**Affiliations:** ^1^Beijing Ophthalmology and Visual Sciences Key Laboratory, Beijing Tongren Eye Center, Beijing Tongren Hospital, Capital Medical University, Beijing, China; ^2^Department of Ophthalmology, Schepens Eye Research Institute of Massachusetts Eye and Ear, Harvard Medical School, Boston, MA, United States; ^3^Department of Ophthalmology, Eye Institute, Eye & ENT Hospital, Fudan University, Shanghai, China

**Keywords:** metabolomics, metabolite profile, glaucoma, retinal ganglion cells, optic neuropathy

## Abstract

Glaucoma is a leading cause of blindness worldwide. It is suggested that primary open angle glaucoma (POAG), the most common form of glaucoma, may be associated with significant metabolic alternations, but the systemic literature review and meta-analysis in the area have been missing. Altered metabolomic profiles in the aqueous humor and plasma may serve as possible biomarkers for early detection or treatment targets. In this article, we performed a systematic meta-analysis of the current literature surrounding the metabolomics of patients with POAG and metabolites associated with the disease. Results suggest several metabolites found to be specifically altered in patients with POAG, suggesting broad generalizability and pathways for future research.

## Introduction

Glaucoma is the leading cause of irreversible blindness characterized by progressive damage of retinal ganglion cells (RGCs) and the optic nerve. It affects nearly 80 million people worldwide, and this number is expected to reach 111.8 million by 2040 (Tham et al., 2014). As the most common type of glaucoma, primary open angle glaucoma (POAG) is a multifactorial neurodegenerative disease, which has been linked to vascular, genetic, anatomical, and immune factors (Zhang et al., 2020). Despite its high prevalence and increasing public health burden, the diagnosis and therapy of POAG present critical unmet medical needs. Patients with POAG are conventionally diagnosed based on clinical and ancillary examinations only if symptoms appear. Elevated intraocular pressure (IOP) is a major and only modifiable risk factor of POAG, although it is neither necessary nor sufficient to cause glaucoma. Current treatment targets solely at lowering IOP. Identification of biomarkers to allow early diagnosis and prompt treatment thus are crucial in preventing permanent and irreversible visual loss of POAG (Heijl et al., 2002; Weinreb et al., 2014).

To date, analysis of transcriptomics, proteomics, and metabolomics have been attempted to uncover the complicated pathogenesis of POAG (Bhattacharya et al., 2013; Takamoto and Araie, 2014; Funke et al., 2016; Shiga et al., 2018). Metabolomics started to develop during the recent decade, providing not only novel biomarkers for diseases but also new insights into the pathophysiology by revealing final downstream products of the whole body system (Schrimpe-Rutledge et al., 2016). It has been utilized to study various eye diseases, including glaucoma, age-related macular degeneration, and diabetic retinopathy (Barbosa-Breda et al., 2018). Previous studies revealed that metabolites of gut microbiota play an important role across the great distance of the human body in mediating neuroinflammation and influencing the perpetuation and progression of neurodegenerative diseases of the central nervous system or the retina (Sharon et al., 2016). It is acknowledged that neuroinflammation driven by both innate and adaptive immunity contributes to the progression of glaucomatous neuron loss; thus, the regulation of which may present a therapeutic target (Chen et al., 2018; Jiang et al., 2020; Tang J. et al., 2020; Tang Y. et al., 2020). The microbial metabolites influence immune homeostasis, including immune cell subsets and their functions (Rooks and Garrett, 2016). For instance, short-chain fatty acids (SCFAs) have been shown to contribute to the counts and functionalities of CD4^+^ regulatory T cells and microglia (Luu et al., 2019), which deeply participate in the pathophysiology of glaucoma (Chen et al., 2018). In such an aspect, metabolites might be critical and promising for the diagnosis and potential treatment of glaucoma.

Currently, correlative studies between specific metabolites and the development of POAG are only beginning to be exploited, including targeted and semi-targeted approaches (Edwards et al., 2014; Burgess et al., 2015; Leruez et al., 2018; Buisset et al., 2019; Yizhen et al., 2019; Kouassi Nzoughet et al., 2020; Myer et al., 2020; Pan et al., 2020). Both approaches yield abundant information on the changes of metabolites and suggest discriminant metabolites involved in steroid biosynthesis, mitochondrial oxidation of energetic substrates, senescence, and polyamine function in the plasma of patients with POAG (Burgess et al., 2015; Leruez et al., 2018; Kouassi Nzoughet et al., 2020). Studies performed till now have created a copious amount of information on the metabolites in aqueous humor and plasma of patients with glaucoma. However, current outcomes are not consistently validated by large sample size and analysis techniques. The present meta-analysis is set up to summarize the metabolomic profiles of POAG to gain further insights into the pathogenesis of the disease.

## Methods

### Search Strategy

A systematic search of the database includes PubMed, Embase, and Web of Science, which were performed to identify metabolomic studies on glaucoma dated up to August 2021. The following terms “glaucoma” and “metabolomics” OR “metabolomic” were used to search for studies in the selected database. The relevant reviews and additional reference lists were also scanned for potential literature. Two independent reviewers conducted a preliminary review of the abstract and results and analyzed the full text to select studies that meet our predefined criteria. The disagreements between the two reviewers were resolved through careful discussion, involving the third reviewer, if necessary, until a consensus was reached.

### Inclusion and Exclusion Criteria

Inclusion criteria included studies focusing on POAG, and the analysis of the metabolites of the aqueous humor or blood plasma using nuclear magnetic resonance or liquid or gas chromatography-mass spectrometry.

The excluded studies are those focused on the mouse or other animal models, or an alternative form of glaucoma, other ocular diseases, or the metabolites of a body fluid other than blood plasma and aqueous humor.

### Quality Assessment

The Newcastle–Ottawa scale (NOS) was used for quality assessment. The NOS contains eight items (nine scores in total), which fit into three categories: selection (four scores), comparability (two scores), and exposure of a case–control study or outcome of a cohort study (three scores). A score of ≥6 indicates good quality.

### Pathway Analysis Process

All metabolites and differentially expressed metabolites (DEMs) were summarized as pooled DEMs for aqueous humor and plasma, respectively. Pathways enrichment analysis of pooled DEMs was conducted using MetaboAnalyst v4.0 and Kyoto Encyclopedia of Genes and Genomes (KEGG) database (Chong et al., 2018).

### Data Extraction Process

The patient data were extracted from the selected studies *via* a standard form: first author, year of publication, country, age of the patient, sex of the patient, sample size, sample material, quality control, and metabolomic analyzing platform. The second reviewer double-checked all data. The included studies reported the outcomes with various forms. To get the same form of the outcomes, the fold change (FC) and standard error (SE) were calculated as follows:

(1) If the median and interquartile range (IQR) are available in the included studies, we estimated mean = median and estimated standard deviation (SD) = IQR/1.35, and then the SE of the log FC was calculated as follows (Lajeunessei, 2011):


$$S\,E\,\left[\log\left(F\,C\right)\right]=S\,E\,\left[\log\left(\frac{m_{1}}{m_{2}}\right)\right]=\sqrt{\frac{s_{1}^{2}}{n_{1}\times m_{1}^{2}}+\frac{s_{2}^{2}}{n_{2}\times m_{2}^{2}}}$$

Where *FC* = *m*_1_/*m*_2_, *m*_*1*_ and *m*_*2*_ are the mean values, *s*_*1*_ and *s*_*2*_ are the SDs, and *n*_*1*_ and *n*_*2*_ are the sample sizes.

(2) If mean and SD are available in the included studies, then the SE of the log FC was directly calculated as the above formula.

(3) If FC and *p*-value or adjust *p*-value are available in the included studies, then the adjust *p-*value (*q-*value) was transferred to *p* as:


p=q×i/N,

Where *i* is the rank and *N* is the total detected metabolites.

Then the SE of the log FC was calculated as follows:


S⁢E⁢[log⁢(F⁢C)]=log⁢(F⁢C)/z

Where *z*-score was calculated from *p*/2 value (one side).

Finally, the log(*FC*), *SE*, *n*_*1*_, and *n*_*2*_ were used to perform the meta-analysis.

### Statistical Analysis

The statistical analysis was performed using Review Manager 5.3. The weighted mean difference (WMD) and 95% confidence interval (CI) were calculated from selected outcomes. A value of *p* < 0.05 was considered statistically significant. Statistical heterogeneity was tested using the chi-squared and *I*^2^ tests. A random-effect meta-regression model was used due to the divergence of the patient population and the metabolite detection methods.

## Results

### Literature Selection

**FIGURE 1:**
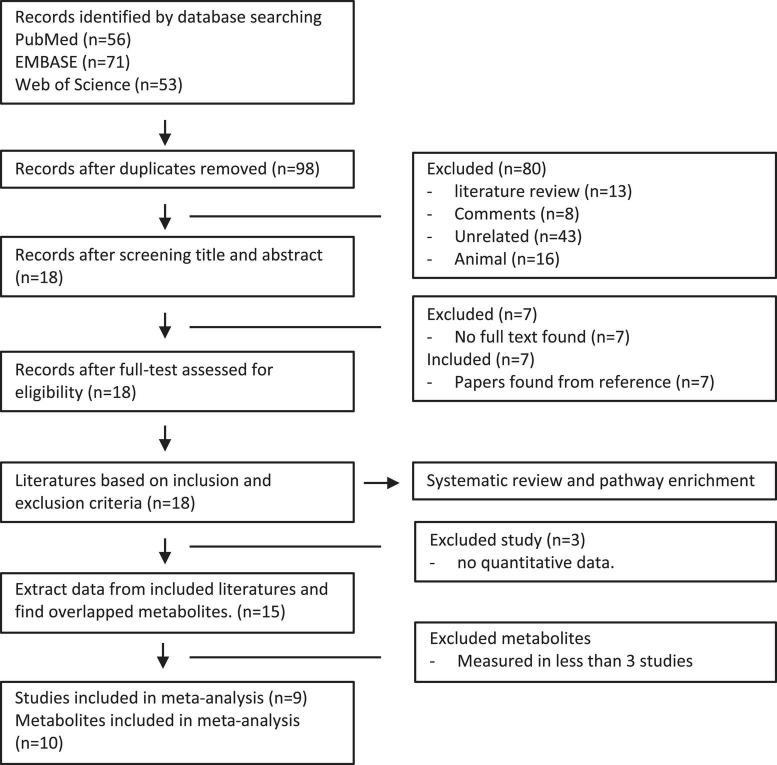
Flow diagram of literature search and study selection in patients with POAG and controls.

We performed a systemic search of databases and literature in PubMed, Embase, and Web of Science using the words “glaucoma” and “metabolomics” or “metabolomic” (Figure 1). Among the 180 reports reviewed independently and in duplicate by two investigators, 82 duplicated databases were excluded. In the remaining 98 studies retrieved, we removed 80 studies that were noted to be literature reviews/comments, analysis in animal models, or unrelated reports. Following the full-text article reviewed thereafter, 7 studies without accessible text were further excluded. After the final addition of 7 references identified through hand searching of citations of all reports, 18 studies met the inclusion criteria in this analysis (Figure 1). The characteristics of the included studies are summarized in Table 1. Among these studies, 7 were analyzed in aqueous humor, 7 were analyzed in plasma, and 4 were analyzed in both. The number of participants in these studies ranged from 12 to 506. Finally, 15 studies have extractable quantitative data for meta-analysis.

**TABLE 1:** Basic information of the studies included in this review and meta-analysis.

First authors (years)	Country	Control	Age (mean ± SD)		Male/Female		Sample size	Sample material	Platform	Quality control
			Ctrl	POAG	Ctrl	POAG	Ctrl/POAG			
Myer et al. (2020)	United States	Cataract	70.71 ± 8.05	73.74 ± 9.07	35/0	20/3	35/23	AH	NMR, IROA	6
Buisset et al. (2019)	France	Cataract	74.69	74.92	14/12	14/12	26/26	AH	LC-MS	7
Barbosa Breda et al. (2020)	Belgium	Cataract	75 ± 8	72 ± 10	13/16	10/17	29/27	AH	NMR	7
Skrzypecki et al. (2021)	Poland	Cataract	73.7 ± 1.8	72.1 ± 1.9	/	/	20/20	AH and Plasma	LC-MS	6
Ghanem et al. (2012)	Egypt	Cataract	61.28 ± 3.23	63.53 ± 4.72	19/16	26/24	35/50	AH and Plasma	LC-MS	7
Ghanem et al. (2011)	Egypt	Cataract	61.3 ± 3.4	62.2 ± 2.5	14/16	18/17	30/35	AH and Plasma	Enzyme-linked immunosorbent assay	7
Cabrerizo et al. (2017)	Spain	Healthy myopia	55.9 ± 7.96	68.8 ± 7.83	6/4	4/6	10/10	AH	LC-MS (lipid)	7
Kotikoski et al. (2002)	Finland	Cataract	77 ± 7	75 ± 8	8/30	8/30	38/38	AH	Immunoassay	7
Tang et al. (2021b)	China	Cataract	65.6 ± 11.32	58.89 ± 14.9	11/14	16/12	25/28	AH and Plasma	LC-MS	7
Leruez et al. (2018)	France	Cataract	73.04	72	15/12	15/21	27/36	Plasma	LC-MS	7
Kouassi Nzoughet et al. (2020)	France	Cataract	73.77	73.06	15/15	17/17	30/34	Plasma	LC-MS	7
Kouassi Nzoughet et al. (2019)	France	Cataract	70.27	64.85	7/8	15/5	15/20	Plasma	LC-MS	7
Umeno et al. (2019)	Japan	Cataract	70.6 ± 10.9	70.4 ± 11.1	36/83	92/106	119/198	Plasma	LC-MS	6
Javadiyan et al. (2012)	Australia	Healthy	76 ± 8.3	78 ± 8.21	135/160	110/101	295/211	Plasma	LC-MS	6
Pulukool et al. (2021)	India	Cataract	/	/	/	/	6/6	AH	LC-MS	6
Pan et al. (2020)	China	Cataract	74.222	72.5	6/10	12/13	16/25	AH	GC-MS	7
Burgess et al. (2015)	United States	Healthy	68.5	67.8	31/41	27/45	72/72	Plasma	LC-MS	7
Gong et al. (2020)	China	Healthy	53.8 ± 7.87	54.77 ± 9.32	14/16	14/16	30/30	Plasma	GC-MS	7

### Common Metabolites and Associated Pathways Identified in Patients With Primary Open Angle Glaucoma

Data from the 18 case–control studies identified 133 metabolites in aqueous humor and 101 in plasma that were uniquely changed in patients with POAG compared to control subjects. The metabolites that were shown to be significantly altered/differentially expressed (DEMs) in either the aqueous humor or plasma of patients with POAG are summarized in Supplementary Tables 1, 2, respectively.

**FIGURE 2:**
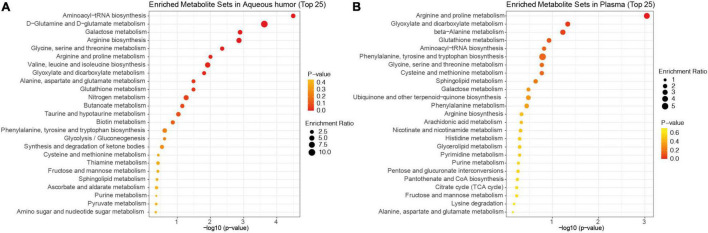
KEGG pathway enrichment analysis of the pooled metabolites. Dot plots of differentially expressed metabolites in the aqueous humor **(A)** and plasma **(B)** of patients with POAG and controls ranked by *p*-value. The bubble size indicates the enriched factor in each pathway and the color bar shows the *p*-value.

We next performed pathway enrichment analysis based on the metabolites pooled from all 18 studies that were found in the aqueous humor and plasma (Figure 2). The top six significantly enriched pathways detected in the aqueous humor of patients with POAG included aminoacyl-tRNA biosynthesis, D-glutamine and D-glutamate metabolism, galactose metabolism, arginine biosynthesis, glycine metabolism, and arginine metabolism (*p* < 0.01). The analysis identified four significantly enriched pathways in the plasma of patients with POAG vs. control subjects, including arginine and proline metabolism, glyoxylate and dicarboxylate metabolism, and beta-alanine metabolism (*p* < 0.05). Thus, arginine metabolism, which is both enriched in aqueous humor and plasma, is the most striking pathway altered in patients with POAG based on previous studies.

### Meta-Analysis of the Metabolites

**FIGURE 3:**
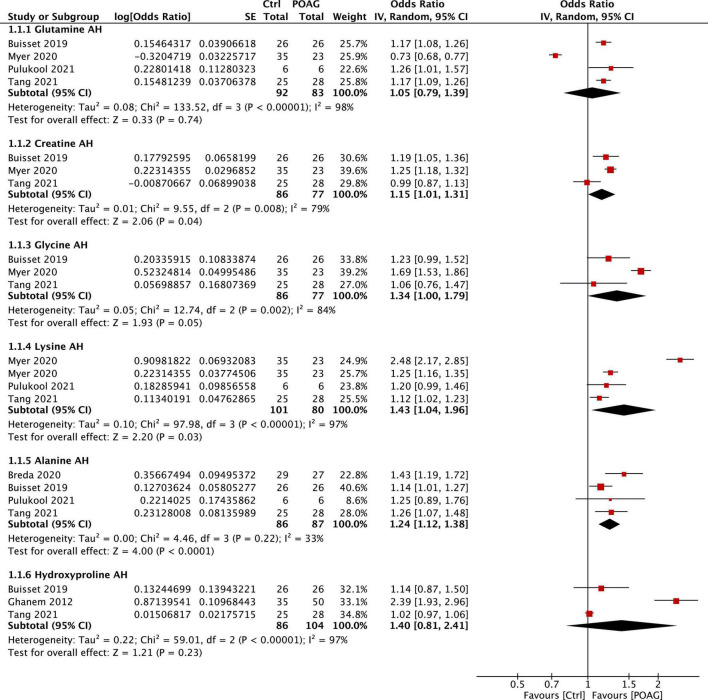
Forest plot of the metabolites in the aqueous humor of patients with POAG compared to controls using a random-effect model. Odds ratio (OR) and 95% confidence intervals (95% CI) were given. The position of the red squares and the horizontal black lines correspond to OR and 95% CI of each study, respectively. The size of the square stands for the weight of the study. The overall OR was displayed by the black diamond, the width of which shows the overall 95% CI of per metabolite. The *I*^2^ and *p*-value for heterogeneity were displayed and the *p*-value for each metabolite is shown after the test for overall effect.

For the meta-analysis, only metabolites reported in at least 3 publications were considered in the present study. Among them, six common metabolites (glutamine, creatine, glycine, lysine, alanine, and hydroxyproline) were noted in 6 studies that analyzed the aqueous humors of control and patients with POAG (Ghanem et al., 2012; Buisset et al., 2019; Barbosa Breda et al., 2020; Myer et al., 2020; Pulukool et al., 2021; Tang et al., 2021b). Since Myer et al. (2020) used two methods [LC–MS/MS and nuclear magnetic resonance (NMR)] to analyze the samples and generated non-consistent outcomes, we treated the outcomes from these two methods as separate datasets. Except for glutamine and hydroxyproline, all of the other four metabolites, creatine (FC = 1.15, 95% CI: 1.01–1.31, *p* = 0.04, *I*^2^ = 79%), glycine (FC = 1.34, 95% CI: 1.00–1.79, *p* = 0.05, *I*^2^ = 84%), lysine (FC = 1.43, 95% CI: 1.04–1.96, *p* = 0.03, *I*^2^ = 97%), and alanine (FC = 1.24, 95% CI: 1.12–1.38, *p* < 0.001, *I*^2^ = 33%), were shown to be significantly higher in the aqueous humors of patients with POAG than that in the control subjects (Figure 3).

**FIGURE 4:**
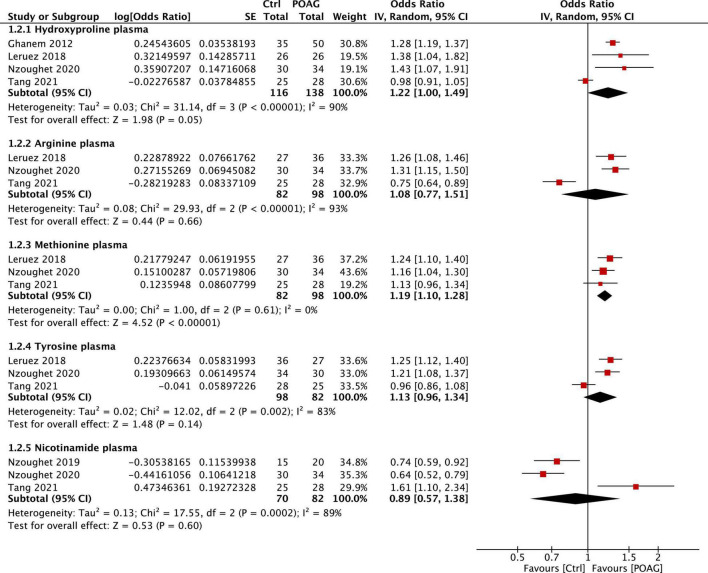
Forest plot of the metabolites in the plasma of patients with POAG compared to controls using a random-effect model. Odds ratio (OR) and 95% confidence intervals (95% CI) were given. The position of the red squares and the horizontal black lines correspond to OR and 95% CI of each study, respectively. The size of the square stands for the weight of the study. The overall OR was displayed by the black diamond, the width of which shows the overall 95% CI of per metabolite. The *I*^2^ and *p-*value for heterogeneity were displayed, and the *p*-value for each metabolite is shown after the test for overall effect.

Significant changes of five plasma metabolites, arginine, methionine, tyrosine, nicotinamide, and hydroxyproline, were reported in patients with POAG compared to control subjects in 5 studies (Ghanem et al., 2012; Leruez et al., 2018; Kouassi Nzoughet et al., 2019, 2020; Tang et al., 2021b). Among them, methionine (FC = 1.19, 95% CI: 1.10–1.28, *p* < 0.001, *I*^2^ = 0%) and hydroxyproline (FC = 1.22, 95% CI: 1.00–1.49, *p* = 0.05, *I*^2^ = 90%) were significantly higher in the plasma of patients with POAG (Figure 4). However, the plasma level of nicotinamide was decreased in patients with POAG in two reports (Kouassi Nzoughet et al., 2019, 2020) but was significantly increased in the other (Tang et al., 2021b). Together, most identified metabolites markers are consistently altered among studies: alanine in aqueous humor and methionine in plasma is the most stable biomarkers for POAG based on this meta-analysis.

## Discussion

**FIGURE 5:**
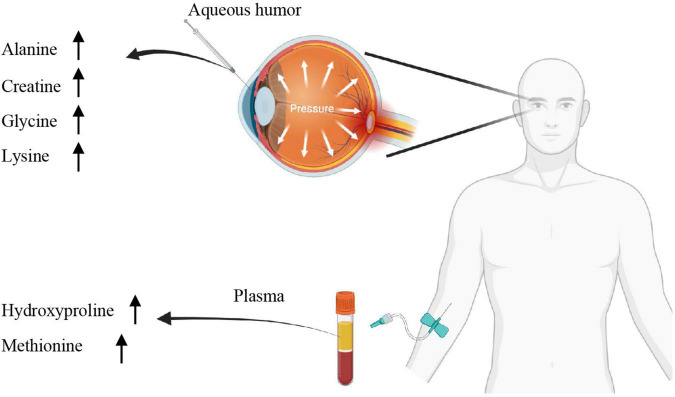
Summary of the dysregulated metabolites.

This systematic review and meta-analyses report a number of metabolites in the aqueous humor and plasma identified using comprehensive high-throughput metabolomics that is prospectively associated with human patients with POAG. Six metabolites in the aqueous humors and five from the plasma were repeatedly shown up in at least three independent studies (Ghanem et al., 2012; Leruez et al., 2018; Buisset et al., 2019; Kouassi Nzoughet et al., 2019, 2020; Barbosa Breda et al., 2020; Myer et al., 2020; Pulukool et al., 2021; Tang et al., 2021b). Main dysregulated metabolites were summarized in Figure 5. These findings might point to possible metabolomic biomarkers and therapeutic targets for glaucoma.

This study reviewed metabolite changes detected in both the aqueous humor and plasma of patients with POAG as determined using various platforms, including nuclear magnetic resonance [NMR or liquid or gas chromatography-mass spectrometry (LC/GC-MS)]. Among these studies, some changes were found to be common in several reports and share similar or overlapping metabolic pathways. For instance, significant upregulation of the metabolites in responding to oxidative stress was reported in both studies by Buisset et al. (2019) and Takayanagi et al. (2020), in agreement with increased oxidative stress in patients with POAG. Mitochondrial energetic substrates were also noted in several of the studies, indicating dysregulated energy metabolism in patients with POAG (Leruez et al., 2018; Kouassi Nzoughet et al., 2020). However, not all changes were consistently revealed among studies, and some led to contradictory readouts. For example, aqueous humor outflow-related metabolites (Myer et al., 2020), trimethylamine and nicotinamide (Kouassi Nzoughet et al., 2020), senescence biomarkers (Leruez et al., 2018), and remodeled cell membrane components (Buisset et al., 2019) were mentioned in separate studies. Glutamine was dramatically decreased in the report by Myer et al. (2020), whereas it was increased in the study by Buisset et al. (2019) and Tang et al. (2021b). In part, reports of common metabolites were usually detected by different groups that employed similar techniques of studies (Leruez et al., 2018; Buisset et al., 2019; Kouassi Nzoughet et al., 2020), whereas opposing findings could be results of different methodologies, disparities of patient populations, or various disease stages studied (IOP variance or POAG at different disease stages) (Kotikoski et al., 2002; Ghanem et al., 2011). Therefore, both the overlapping and opposing findings may offer valuable information reflecting the true pathological processes of the disease.

Lysine is found to be increased in the aqueous humor of patients with POAG. It is an essential amino acid that helps the body produce infection-fighting antibodies, enzymes, hormones, and body tissues. If not used for protein synthesis, lysine is catabolized in mitochondria. Thus, lysine and its degradation product are the reflections of mitochondrial homeostasis. It is reported that lysine supplement boosts the immune responses (Datta et al., 2001). The studies have attested to the importance of the demethylation of lysine 9 of histone H3 (H3K9) in regulating the differentiation of T cells (Scheer and Zaph, 2017) and the proliferation of B cells (Jiang et al., 2019). A combination of L-lysine and L-arginine is one of the most effective supplements for anxiety relief (Lakhan and Vieira, 2010). Not only do lysine and arginine share some metabolism pathways but also turbulence in lysine might also affect arginine, which is found to be significantly increased in the aqueous humor of patients with POAG as described by Myer et al. (2020) and Tang et al. (2021b).

Increased concentrations of creatine in aqueous humor have been verified (Buisset et al., 2019; Myer et al., 2020). Creatine is the product of creatine kinase and ATP, providing energy for muscles including the ciliary body. Elevated creatine levels may lead to increased aqueous humor production and increased IOP. A previous study demonstrated increased concentrations of hydroxyproline, alanine, glutamine, creatine/creatinine, and fatty acids in the aqueous humor of a rat glaucoma model (Mayordomo-Febrer et al., 2015). Studies reported that creatine is neuroprotective to retinal neurons *in vitro* (Sia et al., 2019), and it stabilizes intracellular calcium to protect against hypertonic stress (Alfieri et al., 2006). Creatine might also regulate the immune response by reprogramming macrophage polarization through suppressing IFN-γ-STAT1 signaling (Ji et al., 2019) and T-cell activation *via* T-cell receptor (TCR) signaling (Kazak and Cohen, 2020). Further investigation on creatine and glaucoma neurodegeneration might be needed.

Alanine was found to be consistently increased in the aqueous humor of patients with POAG. Higher alanine concentration was also reported in the retinas of DBA/2J mice (Schuettauf et al., 2007). Alanine can be converted from pyruvate and degraded to pyruvate through transamination by alanine aminotransferase in mitochondria. Thus, an increase of alanine may result from oxidative phosphorylation (OXPHOS) deficiency or dysfunction (Smeitink et al., 2006). Accumulation of L-alanine reduces the production of pyruvate in glycolysis by inhibiting pyruvate kinase and preventing glucose consumption, which is an essential energy source of the retina. This feedback may lead to energy deficiency and further deteriorate neurodegeneration, which is also reported to be the key pathogenesis of glaucoma. Thus, targeting energy deficiency might be another therapeutic perspective for glaucoma (Williams et al., 2017; Tang et al., 2021a).

Hydroxyproline comprises roughly 4% of all the amino acids in the body. Hydroxyproline, proline, and glycine are the major components of collagen, the main building block of connective tissue such as skin, bone, and cartilage. When an injury occurs, hydroxyproline is necessary for repairing tissue damage and fighting against infectious diseases (Li and Wu, 2018). Hydroxyproline is used as a noninvasive oxidative diagnostic marker for bone turnover and liver fibrosis. The generation of hydroxyproline is enhanced in response to oxidative stress as an adaptation mechanism (Wu et al., 2019). Interestingly, hydroxyproline is increased in both the aqueous humor and plasma of patients with POAG, implicating increased oxidative stress under the pathological state of glaucoma.

The essential amino acid L-methionine is a precursor of succinyl-CoA, homocysteine cysteine, creatine, carnitine, and taurine, all critical to eye health. Methionine restriction extends lifespan across different species and exerts beneficial effects on metabolic health and inflammatory responses (Parkhitko et al., 2019). A high serum concentration of methionine is associated with coronary, cerebrovascular, and arterial occlusive diseases (Soares et al., 2017). Recent studies demonstrated that methionine regulates metabolic processes and innate immune responses (Fiil and Gyrd-Hansen, 2014; Martínez et al., 2017) and increases the production of glutathione, taurine, and other metabolites (Martínez et al., 2017). Apart from that, methionine’s derivative feeds into the polyamine, spermidine, and spermidine biosynthesis pathways. A study showed that a diet of spermidine reduced oxidative stress and ameliorates neurodegeneration in a mice glaucoma model (Noro et al., 2015).

Based on the reported changes, we performed pathway enrichment analyses using the pooled metabolites. It is interesting to note that the top five pathways found in the aqueous humor and plasma of patients with POAG overlap a great deal. This similarity maybe expected considering POAG is a chronic neurodegeneration disease that is likely to induce systematic metabolomic changes. Aminoacyl-tRNA biosynthesis (ARSs) and arginine and proline metabolisms are the most striking and common alterations in patients with POAG. ARSs are involved in a broad range of physiological processes (Martinis et al., 1999), and their alteration may lead to changes in cellular viability, activation, and recruitment of immune cells (Nie et al., 2019). Increasing evidence supports that ARSs are involved in both innate and adaptive immune responses (Nie et al., 2019). Arginine metabolism is also known to play an important role in immune regulation (Kim et al., 2018). Therefore, these two pathways may be closely related in function and involved in the pathogenesis of glaucomatous neurodegeneration. Understanding how these metabolites participate in cell signaling and interact with the body’s immune system may provide important insight into the mechanisms and therapeutic targets for glaucoma.

### Limitations and Future Directions

This study underwent thorough vetting of the current literature to create a meta-analysis as holistic as possible. For the included studies, gender, platforms, and patient populations are among the top three intrinsic biases. For instance, no female subject was included in the control group of the report by Myer et al. (2020). Population bias may also exist, as some metabolites (e.g., arginine) in the meta-analysis are based on studies taken from similar regions, such as studies from the same group in France and China. While in a larger dataset study (*n* > 200), no differences in plasma arginine were noted between POAG and control subjects (Javadiyan et al., 2012). In addition, different criteria for control groups might also potentially affect the significance of metabolites. Some studies, albeit fit our inclusion criteria, lacked available and formatted data that could be incorporated in this meta-analysis. Incomplete raw datasets prohibit the detection of significant changes through meta-analysis. While lipids are the main component in metabolomics, inaccurate naming and labeling have made it difficult to analyze the data. In short, changes found in the aqueous humor seem to be more consistent compared to plasma metabolite data, possibly due to the high sensitivity of metabolomics, which can be affected by many factors, including diets and populations, and tissue types and sample processing (Stevens et al., 2019). A broader population and geographic locations in both healthy control subjects should also be included in the future. In the meantime, a dataset with minimized intrinsic bias and available format will provide benefits for the investigation of biomarkers and the pathology of glaucoma.

## Data Availability Statement

The datasets presented in the study are included in the article, further inquiries can be directed to the corresponding author/s.

## Author Contributions

DC and XS contributed to the conception and design of the study. YT and SS searched for the databases. YT analyzed the data. YT, SS, K-SC, and DC wrote the manuscript. All authors contributed to manuscript revision, read, and approved the submitted version.

## Conflict of Interest

DC is a consultant for Boston Pharmaceuticals, FireCyte Therapeutics, i-Lumen Scientific, and PriMed. K-SC is a consultant for SunRegen. XS is a consultant for Rimonci, BELKIN Vision, AffaMed, NovaSight, Ocumension, and KBI. DC, K-SC, and XS are inventors on patents and patents application related to glaucoma. The remaining authors declare that the research was conducted in the absence of any commercial or financial relationships that could be construed as a potential conflict of interest.

## Publisher’s Note

All claims expressed in this article are solely those of the authors and do not necessarily represent those of their affiliated organizations, or those of the publisher, the editors and the reviewers. Any product that may be evaluated in this article, or claim that may be made by its manufacturer, is not guaranteed or endorsed by the publisher.

## References

[B1] Alfieri R. R. Bonelli M. A. Cavazzoni A. Brigotti M. Fumarola C. Sestili P. (2006). Creatine as a compatible osmolyte in muscle cells exposed to hypertonic stress. *J. Physiol.* 576(Pt 2), 391–401. 10.1113/jphysiol.2006.115006 16873409 PMC1890352

[B2] Barbosa Breda J. Croitor Sava A. Himmelreich U. Somers A. Matthys C. Rocha Sousa A. (2020). Metabolomic profiling of aqueous humor from glaucoma patients - The metabolomics in surgical ophthalmological patients (MISO) study. *Exp. Eye Res.* 201:108268. 10.1016/j.exer.2020.108268 33011236

[B3] Barbosa-Breda J. Himmelreich U. Ghesquière B. Rocha-Sousa A. Stalmans I. (2018). Clinical Metabolomics and Glaucoma. *Ophthalmic. Res.* 59 1–6. 10.1159/000479158 28858875

[B4] Bhattacharya S. K. Lee R. K. Grus F. H. Group S. A. (2013). Molecular biomarkers in glaucoma. *Invest Ophthalmol. Vis. Sci.* 54 121–131. 10.1167/iovs.12-11067 23297392 PMC3544416

[B5] Buisset A. Gohier P. Leruez S. Muller J. Amati-Bonneau P. Lenaers G. (2019). Metabolomic Profiling of Aqueous Humor in Glaucoma Points to Taurine and Spermine Deficiency: findings from the Eye-D Study. *J. Proteome Res.* 18 1307–1315. 10.1021/acs.jproteome.8b00915 30701980

[B6] Burgess L. G. Uppal K. Walker D. I. Roberson R. M. Tran V. Parks M. B. (2015). Metabolome-Wide Association Study of Primary Open Angle Glaucoma. *Invest. Ophthalmol. Vis. Sci.* 56 5020–5028. 10.1167/iovs.15-16702 26230767 PMC4525636

[B7] Cabrerizo J. Urcola J. A. Vecino E. (2017). Changes in the Lipidomic Profile of Aqueous Humor in Open-Angle Glaucoma. *J. Glaucoma* 26 349–355. 10.1097/IJG.0000000000000603 28221327

[B8] Chen H. Cho K. S. Vu T. H. K. Shen C. H. Kaur M. Chen G. (2018). Commensal microflora-induced T cell responses mediate progressive neurodegeneration in glaucoma. *Nat. Commun.* 9:3209. 30097565 10.1038/s41467-018-05681-9 PMC6086830

[B9] Chong J. Soufan O. Li C. Caraus I. Li S. Bourque G. (2018). MetaboAnalyst 4.0: towards more transparent and integrative metabolomics analysis. *Nucleic Acids Res.* 46 W486–W494. 10.1093/nar/gky310 29762782 PMC6030889

[B10] Datta D. Bhinge A. Chandran V. (2001). Lysine: Is it worth more? *Cytotechnology* 36 3–32. 10.1023/A:1014097121364 19003311 PMC3449675

[B11] Edwards G. Aribindi K. Guerra Y. Lee R. K. Bhattacharya S. K. (2014). Phospholipid profiles of control and glaucomatous human aqueous humor. *Biochimie* 101 232–247. 10.1016/j.biochi.2014.01.020 24561385 PMC3995849

[B12] Fiil B. K. Gyrd-Hansen M. (2014). Met1-linked ubiquitination in immune signalling. *FEBS J.* 281 4337–4350. 10.1111/febs.12944 25060092 PMC4286102

[B13] Funke S. Perumal N. Beck S. Gabel-Scheurich S. Schmelter C. Teister J. (2016). Glaucoma related Proteomic Alterations in Human Retina Samples. *Sci. Rep.* 6:29759. 10.1038/srep29759 27425789 PMC4947915

[B14] Ghanem A. A. Elewa A. M. Arafa L. F. (2011). Endothelin-1 and nitric oxide levels in patients with glaucoma. *Ophthalmic. Res.* 46 98–102. 10.1159/000323584 21282966

[B15] Ghanem A. A. Mady S. M. El awady H. E. Arafa L. F. (2012). Homocysteine and hydroxyproline levels in patients with primary open-angle glaucoma. *Curr. Eye Res.* 37 712–718. 10.3109/02713683.2012.669512 22458818

[B16] Gong H. Zhang S. Li Q. Zuo C. Gao X. Zheng B. (2020). Gut microbiota compositional profile and serum metabolic phenotype in patients with primary open-angle glaucoma. *Exp. Eye Res.* 191:107921. 10.1016/j.exer.2020.107921 31917963

[B17] Heijl A. Leske M. C. Bengtsson B. Hyman L. Hussein M. Group E. M. G. T. (2002). Reduction of intraocular pressure and glaucoma progression: results from the Early Manifest Glaucoma Trial. *Arch. Ophthalmol.* 120 1268–1279. 10.1001/archopht.120.10.1268 12365904

[B18] Javadiyan S. Burdon K. P. Whiting M. J. Abhary S. Straga T. Hewitt A. W. (2012). Elevation of serum asymmetrical and symmetrical dimethylarginine in patients with advanced glaucoma. *Invest Ophthalmol. Vis. Sci.* 53 1923–1927. 10.1167/iovs.11-8420 22395885

[B19] Ji L. Zhao X. Zhang B. Kang L. Song W. Zhao B. (2019). Slc6a8-Mediated Creatine Uptake and Accumulation Reprogram Macrophage Polarization via Regulating Cytokine Responses. *Immunity* 51 272–284. 10.1016/j.immuni.2019.06.007 31399282

[B20] Jiang S. Kametani M. Chen D. F. (2020). Adaptive Immunity: new Aspects of Pathogenesis Underlying Neurodegeneration in Glaucoma and Optic Neuropathy. *Front. Immunol.* 11:65. 10.3389/fimmu.2020.00065 32117239 PMC7031201

[B21] Jiang Y. Li C. Wu Q. An P. Huang L. Wang J. (2019). Iron-dependent histone 3 lysine 9 demethylation controls B cell proliferation and humoral immune responses. *Nat. Commun.* 10:2935. 10.1038/s41467-019-11002-5 31270335 PMC6610088

[B22] Kazak L. Cohen P. (2020). Creatine metabolism: energy homeostasis, immunity and cancer biology. *Nat. Rev. Endocrinol.* 16 421–436. 10.1038/s41574-020-0365-5 32493980

[B23] Kim S. H. Roszik J. Grimm E. A. Ekmekcioglu S. (2018). Impact of l-Arginine Metabolism on Immune Response and Anticancer Immunotherapy. *Front. Oncol.* 8:67. 10.3389/fonc.2018.00067 29616189 PMC5864849

[B24] Kotikoski H. Moilanen E. Vapaatalo H. Aine E. (2002). Biochemical markers of the L-arginine-nitric oxide pathway in the aqueous humour in glaucoma patients. *Acta Ophthalmol. Scand.* 80 191–195. 10.1034/j.1600-0420.2002.800214.x 11952488

[B25] Kouassi Nzoughet J. Chao de la Barca J. M. Guehlouz K. Leruez S. Coulbault L. Allouche S. (2019). Nicotinamide Deficiency in Primary Open-Angle Glaucoma. *Invest. Ophthalmol. Vis. Sci.* 60 2509–2514. 10.1167/iovs.19-27099 31185090

[B26] Kouassi Nzoughet J. Guehlouz K. Leruez S. Gohier P. Bocca C. Muller J. (2020). A Data Mining Metabolomics Exploration of Glaucoma. *Metabolites* 10:2. 10.3390/metabo10020049 32012845 PMC7074047

[B27] Lajeunessei M. J. (2011). On the meta-analysis of response ratios for studies with correlated and multi-group designs. *Ecology* 92 2049–2055. 10.1890/11-0423.1 22164829

[B28] Lakhan S. E. Vieira K. F. (2010). Nutritional and herbal supplements for anxiety and anxiety-related disorders: systematic review. *Nutr. J.* 9:42. 10.1186/1475-2891-9-42 20929532 PMC2959081

[B29] Leruez S. Marill A. Bresson T. de Saint Martin G. Buisset A. Muller J. (2018). A Metabolomics Profiling of Glaucoma Points to Mitochondrial Dysfunction, Senescence, and Polyamines Deficiency. *Invest. Ophthalmol. Vis. Sci.* 59 4355–4361. 10.1167/iovs.18-24938 30193307

[B30] Li P. Wu G. (2018). Roles of dietary glycine, proline, and hydroxyproline in collagen synthesis and animal growth. *Amino Acids* 50 29–38. 10.1007/s00726-017-2490-6 28929384

[B31] Luu M. Pautz S. Kohl V. Singh R. Romero R. Lucas S. (2019). The short-chain fatty acid pentanoate suppresses autoimmunity by modulating the metabolic-epigenetic crosstalk in lymphocytes. *Nat. Commun.* 10:760. 10.1038/s41467-019-08711-2 30770822 PMC6377655

[B32] Martínez Y. Li X. Liu G. Bin P. Yan W. Más D. (2017). The role of methionine on metabolism, oxidative stress, and diseases. *Amino Acids* 49 2091–2098. 10.1007/s00726-017-2494-2 28929442

[B33] Martinis S. A. Plateau P. Cavarelli J. Florentz C. (1999). Aminoacyl-tRNA synthetases: a family of expanding functions. Mittelwihr, France, October 10-15, 1999. *EMBO J* 18 4591–4596. 10.1093/emboj/18.17.4591 10469639 PMC1171533

[B34] Mayordomo-Febrer A. López-Murcia M. Morales-Tatay J. M. Monleón-Salvado D. Pinazo-Durán M. D. (2015). Metabolomics of the aqueous humor in the rat glaucoma model induced by a series of intracamerular sodium hyaluronate injection. *Exp. Eye Res.* 131 84–92. 10.1016/j.exer.2014.11.012 25479046

[B35] Myer C. Perez J. Abdelrahman L. Mendez R. Khattri R. B. Junk A. K. (2020). Differentiation of soluble aqueous humor metabolites in primary open angle glaucoma and controls. *Exp. Eye Res.* 194:108024. 10.1016/j.exer.2020.108024 32246983 PMC7229990

[B36] Nie A. Sun B. Fu Z. Yu D. (2019). Roles of aminoacyl-tRNA synthetases in immune regulation and immune diseases. *Cell Death Dis.* 10:901. 10.1038/s41419-019-2145-5 31780718 PMC6883034

[B37] Noro T. Namekata K. Azuchi Y. Kimura A. Guo X. Harada C. (2015). Spermidine Ameliorates Neurodegeneration in a Mouse Model of Normal Tension Glaucoma. *Invest Ophthalmol. Vis. Sci.* 56 5012–5019. 10.1167/iovs.15-17142 26230766

[B38] Pan C. W. Ke C. Chen Q. Tao Y. J. Zha X. Zhang Y. P. (2020). Differential metabolic markers associated with primary open-angle glaucoma and cataract in human aqueous humor. *BMC Ophthalmol.* 20:183. 10.1186/s12886-020-01452-7 32375707 PMC7203853

[B39] Parkhitko A. A. Jouandin P. Mohr S. E. Perrimon N. (2019). Methionine metabolism and methyltransferases in the regulation of aging and lifespan extension across species. *Aging Cell* 18 e13034. 10.1111/acel.13034 31460700 PMC6826121

[B40] Pulukool S. K. Bhagavatham S. K. S. Kannan V. Sukumar P. Dandamudi R. B. Ghaisas S. (2021). Elevated dimethylarginine, ATP, cytokines, metabolic remodeling involving tryptophan metabolism and potential microglial inflammation characterize primary open angle glaucoma. *Sci. Rep.* 11:9766. 33963197 10.1038/s41598-021-89137-z PMC8105335

[B41] Rooks M. G. Garrett W. S. (2016). Gut microbiota, metabolites and host immunity. *Nat. Rev. Immunol.* 16 341–352. 10.1038/nri.2016.42 27231050 PMC5541232

[B42] Scheer S. Zaph C. (2017). The Lysine Methyltransferase G9a in Immune Cell Differentiation and Function. *Front. Immunol.* 8:429. 10.3389/fimmu.2017.00429 28443098 PMC5387087

[B43] Schrimpe-Rutledge A. C. Codreanu S. G. Sherrod S. D. McLean J. A. (2016). Untargeted Metabolomics Strategies-Challenges and Emerging Directions. *J. Am. Soc. Mass Spectrom.* 27 1897–1905. 10.1007/s13361-016-1469-y 27624161 PMC5110944

[B44] Schuettauf F. Thaler S. Bolz S. Fries J. Kalbacher H. Mankowska A. (2007). Alterations of amino acids and glutamate transport in the DBA/2J mouse retina; possible clues to degeneration. *Graefes Arch. Clin. Exp. Ophthalmol.* 245 1157–1168. 10.1007/s00417-006-0531-z 17226020

[B45] Sharon G. Sampson T. R. Geschwind D. H. Mazmanian S. K. (2016). The Central Nervous System and the Gut Microbiome. *Cell* 167 915–932. 10.1016/j.cell.2016.10.027 27814521 PMC5127403

[B46] Shiga Y. Akiyama M. Nishiguchi K. M. Sato K. Shimozawa N. Takahashi A. (2018). Genome-wide association study identifies seven novel susceptibility loci for primary open-angle glaucoma. *Hum. Mol. Genet.* 27 1486–1496. 10.1093/hmg/ddy053 29452408 PMC6251544

[B47] Sia P. I. Wood J. P. M. Chidlow G. Casson R. (2019). Creatine is Neuroprotective to Retinal Neurons In Vitro But Not In Vivo. *Invest. Ophthalmol. Vis. Sci.* 60 4360–4377. 10.1167/iovs.18-25858 31634394

[B48] Skrzypecki J. Izdebska J. Kamińska A. Badowska J. Przybek-Skrzypecka J. Bombuy J. (2021). Glaucoma patients have an increased level of trimethylamine, a toxic product of gut bacteria, in the aqueous humor: a pilot study. *Int. Ophthalmol.* 41 341–347. 10.1007/s10792-020-01587-y 32914277 PMC7840632

[B49] Smeitink J. A. Zeviani M. Turnbull D. M. Jacobs H. T. (2006). Mitochondrial medicine: a metabolic perspective on the pathology of oxidative phosphorylation disorders. *Cell Metab.* 3 9–13. 10.1016/j.cmet.2005.12.001 16399500

[B50] Soares M. S. Oliveira P. S. Debom G. N. da Silveira, Mattos B. Polachini C. R. (2017). Chronic administration of methionine and/or methionine sulfoxide alters oxidative stress parameters and ALA-D activity in liver and kidney of young rats. *Amino Acids* 49 129–138. 10.1007/s00726-016-2340-y 27718024

[B51] Stevens V. L. Hoover E. Wang Y. Zanetti K. A. (2019). Pre-Analytical Factors that Affect Metabolite Stability in Human Urine, Plasma, and Serum: a Review. *Metabolites* 9:8. 10.3390/metabo9080156 31349624 PMC6724180

[B52] Takamoto M. Araie M. (2014). Genetics of primary open angle glaucoma. *Jpn J. Ophthalmol.* 58 1–15. 24258795 10.1007/s10384-013-0286-0

[B53] Takayanagi Y. Takai Y. Kaidzu S. Tanito M. (2020). Evaluation of Redox Profiles of the Serum and Aqueous Humor in Patients with Primary Open-Angle Glaucoma and Exfoliation Glaucoma. *Antioxidants* 9:12. 10.3390/antiox9121305 33352680 PMC7765903

[B54] Tang J. Tang Y. Yi I. Chen D. F. (2020). The role of commensal microflora-induced T cell responses in glaucoma neurodegeneration. *Prog. Brain Res.* 256 79–97. 10.1016/bs.pbr.2020.06.002 32958216

[B55] Tang Y. Fang W. Xiao Z. Song M. Zhuang D. Han B. (2021a). Nicotinamide ameliorates energy deficiency and improves retinal function in Cav-1. *J. Neurochem.* 157 550–560. 10.1111/jnc.15266 33305362

[B56] Tang Y. Pan Y. Chen Y. Kong X. Chen J. Zhang H. (2021b). Metabolomic Profiling of Aqueous Humor and Plasma in Primary Open Angle Glaucoma Patients Points Towards Novel Diagnostic and Therapeutic Strategy. *Front. Pharmacol.* 12:621146. 10.3389/fphar.2021.621146 33935712 PMC8080440

[B57] Tang Y. Xiao Z. Pan L. Zhuang D. Cho K. S. Robert K. (2020). Therapeutic Targeting of Retinal Immune Microenvironment With CSF-1 Receptor Antibody Promotes Visual Function Recovery After Ischemic Optic Neuropathy. *Front. Immunol.* 11:585918. 10.3389/fimmu.2020.585918 33281816 PMC7691249

[B58] Tham Y.-C. Li X. Wong T. Y. Quigley H. A. Aung T. Cheng C.-Y. (2014). Global prevalence of glaucoma and projections of glaucoma burden through 2040: a systematic review and meta-analysis. *Ophthalmology* 121 2081–2090. 10.1016/j.ophtha.2014.05.013 24974815

[B59] Umeno A. Tanito M. Kaidzu S. Takai Y. Horie M. Yoshida Y. (2019). Comprehensive measurements of hydroxylinoleate and hydroxyarachidonate isomers in blood samples from primary open-angle glaucoma patients and controls. *Sci. Rep.* 9:2171. 10.1038/s41598-018-36952-6 30778084 PMC6379359

[B60] Weinreb R. N. Aung T. Medeiros F. A. (2014). The pathophysiology and treatment of glaucoma: a review. *JAMA* 311 1901–1911. 10.1001/jama.2014.3192 24825645 PMC4523637

[B61] Williams P. A. Harder J. M. Foxworth N. E. Cochran K. E. Philip V. M. Porciatti V. (2017). Vitamin B3 modulates mitochondrial vulnerability and prevents glaucoma in aged mice. *Science* 355 756–760. 10.1126/science.aal0092 28209901 PMC5408298

[B62] Wu Z. Hou Y. Dai Z. Hu C. A. Wu G. (2019). Metabolism, Nutrition, and Redox Signaling of Hydroxyproline. *Antioxid. Redox Signal.* 30 674–682. 10.1089/ars.2017.7338 28934858

[B63] Yizhen T. Jihong W. Xinghuai S. (2019). Advances in metabolomics research of glaucoma. *Internat. Rev. Ophthal.* 43 294–299.

[B64] Zhang N. Wang J. Chen B. Li Y. Jiang B. (2020). Prevalence of Primary Angle Closure Glaucoma in the Last 20 Years: a Meta-Analysis and Systematic Review. *Front. Med.* 7:624179. 10.3389/fmed.2020.624179 33537335 PMC7847989

